# Construction Notes

**Published:** 1897-06-12

**Authors:** 


### CONSTRUCTION NOTES.

**Bristol Dispensary.—New Branch at Bedminster.**

A new branch dispensary in connection with the Central Bristol Institution will soon now be opened. This was designed by Mr. W. V. Gough, the contractor being Mr. George Humphreys. The building stands back from the road and is of red brick, with freestone designs and Brossly tiles. The panel bearing the name Bristol Dispensary is also of freestone. The dispensary is approached by an incline which rises to the central doorway. This opens into a hall seven feet in width, and on the one side is a large general waiting-room, to which the dispensary is attached, while on the other there is a second waiting-room connected with the doctors' consulting-rooms. The first floor is arranged as a residence for the dispenser, and the basement is used as a store and also for the heating apparatus. The site cost £450, and the report shows that on the building £575 has already been spent, and that £1,400 more will be required. An appeal will be made for the sum required. The branch dispensary is to meet the needs of the rapidly growing town of Bristol, no part of which has shown a more rapid increase of late years than the locality where the new dispensary has been erected.

**Proposed House of Mercy and Convalescent Home for Dundee.**

The site of this building is on the vacant ground to the north of the present convent buildings and within the boundary walls. The house of mercy as planned will have sleeping accommodation for 32 girls, having large refectory, school-room, recreation-room, waiting-room, and registry office, with kitchen, &c., on the ground floor, and two large dormitories, a large bed-room, and ample bath-room and napery accommodation on the first floor. A special feature of this block will be a large, convenient, and well-appointed laundry. The convalescent home will have dining-room, parlour, and work-room on the ground floor, and five bed-rooms with bath-room accommodation on the first floor. To economise space and service, the kitchen premises have been so designed as to be convenient and central for both buildings, which, although conjoined, are entered by separate doors. The building is estimated to cost about £6,000.

